# Morphological and molecular characterization of *Henneguya cardii* n. sp. (Cnidaria: Myxosporea) from the bulbus arteriosus of European seabass *Dicentrarchus labrax* (Teleostei: Moronidae)

**DOI:** 10.1017/S0031182024001112

**Published:** 2024-09

**Authors:** Luis F. Rangel, Ricardo Severino, Maria J. Santos, Sónia Rocha

**Affiliations:** 1 Interdisciplinary Centre of Marine and Environmental Research (CIIMAR/CIMAR), Laboratory of Animal Parasitology and Pathology, University of Porto, Matosinhos, Portugal; 2 CIIMAR, Departamento de Biologia, Faculdade de Ciências, Universidade do Porto, Porto, Portugal; 3 Instituto de Investigação e Inovação em Saúde (i3S), University of Porto, Porto, Portugal; 4 Institute of Biomedical Sciences Abel Salazar (ICBAS), University of Porto, Porto, Portugal

**Keywords:** aquaculture, heart infection, Myxobolidae, parasite, phylogeny

## Abstract

A new species of Myxobolidae, *Henneguya cardii* n. sp., is described infecting the European seabass *Dicentrarchus labrax*, a fish of high commercial value intensively cultivated in southern Europe. *Henneguya cardii* n. sp. was found in the bulbus arteriosus and spleen with a prevalence of infection of 13.5%. In the heart, it forms irregular whitish plasmodia measuring 1 mm in size. Mature myxospores are broadly obovoid in frontal view and ellipsoidal in lateral view, with 2 equal caudal appendages. Polar capsules are ovoid and symmetric, with 3–4 polar tubule coils. Myxospores measure 10.2 ± 0.6 (8.8–11.6) μm in length, 8.0 ± 0.7 (5.3–8.8) μm in width and 5.6 ± 0.2 (5.1–6.4) μm in thickness. Caudal appendages are 36.6 ± 3.2 (27.4–42.9) μm long. Total spore length is 47.6 ± 3.2 (41.2–53.2) μm. Polar capsules measure 4.0 ± 0.2 (3.4–4.6) by 2.2 ± 0.1 (1.9–2.6) μm. Small subunit ribosomal RNA-based tree topologies position *H. cardii* n. sp. within a lineage of marine myxobolids that is mostly comprised of other *Henneguya* species. Host-relatedness is reinforced as the main evolutionary driver for myxobolids, with the positioning of *H. cardii* n. sp. further suggesting tissue tropism as another important evolutionary driver for marine heart infecting *Henneguya*. Nonetheless, the inner complexity of this lineage suggests that identification of the evolutionary patterns driving its phylogeny will require discovery of the true diversity of marine myxobolids.

## Graphical abstract

**Figure:**
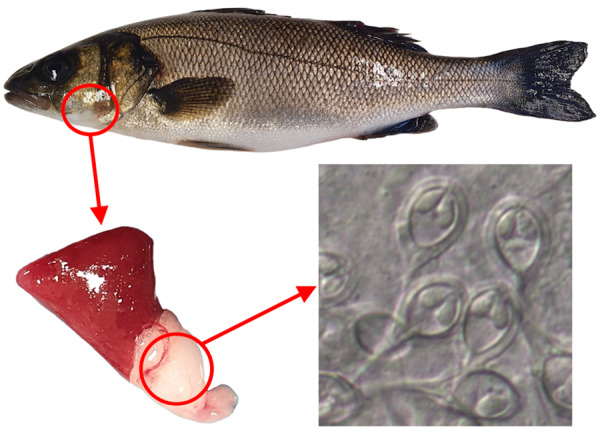

## Introduction

Myxosporeans are cnidarian parasites with a complex 2-host life cycle that involves annelid worms as definitive hosts and usually fish as intermediate hosts (Lom and Dyková, 2006). Several myxosporeans cause severe fish diseases that result in considerable economic losses in the fishing and aquaculture industries (Pote *et al*., 2000; Kent *et al*., 2001; MacKenzie *et al*., 2005; Feist and Longshaw, 2006). The genus *Henneguya* Thélohan, 1892 of the family Myxobolidae Thélohan, 1892, is one of the most diversified myxosporean taxa, comprising more than 250 species described worldwide (Rangel *et al*., 2023). *Henneguya* are predominantly histozoic parasites of freshwater fishes, occurring less frequently in brackish/marine hosts. The gills are the main site of infection for these parasites, but they have also been reported from the epidermis, heart, kidneys, intestine, fins, urinary bladder, brain, eyes, gallbladder, liver, gonads, swim bladder, abdominal cavity, cartilage, spleen, among other organs and tissues (Eiras, 2002; Eiras and Adriano, 2012; Rangel *et al*., 2023).

The European seabass, *Dicentrarchus labrax* (Linnaeus, 1758) (Moronidae), is an important commercial fish intensively cultured in Portugal and in the Mediterranean (Rocha *et al*., 2022). Several myxosporeans have been described or reported from this fish host: *Sphaerospora testicularis* Sitjà-Bobadilla and Álvarez-Pellitero, 1990 from the testis; *Kudoa dicentrarchi* (Sitjà-Bobadilla and Álvarez-Pellitero, 1992) from the connective tissue of all organs; *Ceratomyxa labracis* Sitjà-Bobadilla and Álvarez-Pellitero, 1993 and *Ceratomyxa diplodae* Lubat, Radujković, Marques and Bouix, 1989 from the gallbladder; *Ortholinea labracis* Rangel, Rocha, Casal, Castro, Severino, Azevedo, Cavaleiro and Santos, 2017 from the urinary bladder and a *Myxobilatus* sp. from the kidney and urinary bladder (Lubat *et al*., 1989; Sitjà-Bobadilla and Álvarez-Pellitero, 1990, 1992, 1993; Santos, 1996; Rocha *et al*., 2016; Rangel *et al*., 2017; Casal *et al*., 2019). Except for the latter, all these species have been reported to occur in stocks of *D. labrax* cultured in southern Portuguese fish farms (Rangel *et al*., 2016, 2017; Rocha *et al*., 2016; Castro *et al*., 2018). Two other myxosporeans, originally described from fish other than European seabass, have also been reported from this host in other geographic locations: *Enteromyxum leei* (Diamant, Lom and Dyková, 1994) in the gut (Palenzuela *et al*., 2002; Fioravanti *et al*., 2006), and *Kudoa iwatai* Egusa and Shiomitsu, 1983 in the muscle and other organs (Diamant *et al*., 2005). Additionally, an unnamed *Henneguya* species infecting the bulbus arteriosus of *D. labrax* has been reported from St. Gilla lagoon in Sardinia (Italy) (Culurgioni *et al*., 2010). A novel *Henneguya* species is described from the bulbus arteriosus of European seabass cultured in a southern Portuguese fish farm, based on a combination of morphological, biological and molecular data. This is the first description of a *Henneguya* species from Portugal, despite numerous myxobolids having been reported from Portuguese freshwater and estuarine environments (Saraiva and Chubb, 1989; Cruz *et al*., 1998, 2000; Molnár *et al*., 2012; Rocha *et al*., 2019*a*, 2019*b*; Guimarães *et al*., 2023). This species was discovered in the course of research projects on Myxozoa and Coccidia parasites in farmed fish species.

## Materials and methods

### Sampling of European seabass

Ninety-six specimens of European seabass (*D. labrax*) were obtained from a fish farm located in the Alvor estuary, Atlantic coast (37°08′N, 8°37′W), Portimão, in Algarve, southern Portugal, from May 2019 to July 2021. Fresh specimens were weighted (g) and measured (cm) prior to being dissected for performing a myxosporean survey of internal organs. Due to mandatory confinements related to the COVID-19 pandemic, fish samplings could not be performed periodically, rendering it impossible to determine the seasonal prevalence of infections.

### Myxosporean survey and light microscopy analyses

Several organs, including the heart, spleen, gills, liver, kidneys, gallbladder, intestine and urinary bladder were examined macroscopically using a Zeiss Stemi 2000-C binocular microscope (Grupo Taper, Sintra, Portugal) to detect structural changes or the presence of plasmodia. Microscopic examination of fresh tissue smears was performed using a Zeiss Axiophot microscope (Grupo Taper). Fresh mature myxospores were measured and photographed using an optical microscope equipped with a Zeiss Axiocam digital camera Icc3 and Zeiss Axiovision 4.6.3 software (Grupo Taper). Measurements were performed as recommended by Lom and Arthur (1989) for *Henneguya* myxospores and are presented as mean ± s.d. (range), followed by the number of myxospores measured for each character.

### DNA extraction, amplification and sequencing

DNA from ethanol-preserved myxospores of 2 isolates from 2 individual hosts (R1H and R2H) was separately extracted using the commercial kit GenElute™ Mammalian Genomic DNA Miniprep Kit (Sigma-Aldrich, Merck Life Science S.L.U., Algés, Portugal), following the manufacturer's instructions. Amplification of the parasite's small subunit ribosomal RNA (SSU rDNA) gene was achieved through the polymerase chain reaction (PCR) using a combination of universal eukaryotic primers and myxozoan-specific primers (Table 1). PCRs were performed in 25 μL reactions containing 2.5 mm of MgCl_2_, 1×Taq DNA polymerase reaction buffer, 1.25 U of Taq polymerase (NZYTaq II DNA polymerase, NZYTech, Lisbon, Portugal), 0.2 mm deoxyribonucleotide triphosphates (dNTPs) and 0.2 μm of each primer (NZYTech). Amplifications were performed in a Bio-Rad-MJ Mini Gradient Thermal Cycler with initial denaturation at 95°C for 3 min, followed by 35 cycles of denaturation at 94°C for 35 s, annealing at 55°C for 35 s, extension at 72°C for 45 s and a final extension at 72°C for 7 min. Five microliters of the PCR products were screened in a 1% agarose gel in TAE buffer (NZYTech) to confirm amplification. PCR products were purified, and sequenced using the same PCR primers (Table 1) by STAB VIDA (Caparica, Portugal) in a BigDye Terminator v3.1 from the Applied Biosystems Kit and run on an ABI3730XL DNA analyser (Applied Biosystems, STAB VIDA Lda., Caparica, Portugal).

**Table 1.** Primers used to amplify and sequencing the SSU rDNA of *Henneguya* myxospores found infecting the bulbus arteriosus of *Dicentrarchus labrax*

Name	Sequence (5′–3′)	Used with	Source
18e	CTGGTTGATCCTGCCAGT	ACT3R, ACT1R	Hillis and Dixon (1991)
18R	CTACGGAAACCTTGTTACG	ACT3F	Whipps *et al*. (2003)
ERIB1	ACCTGGTTGATCCTGCCAG	ACT1R	Barta *et al*. (1997)
ACT3f	CATGGAACGAACAAT	18R	Hallett and Diamant (2001)
ACT3R	ATTGTTCGTTCCATG	18e	Hallett and Diamant (2001)
ACT1R	AATTTCACCTCTCGCTGCCA	18e, ERIB1	Hallett and Diamant (2001)

### Phylogenetic analysis

The partial sequences obtained from the PCR products of the 2 isolates were separately assembled using ClustalW in MEGA X software (Nei and Kumar, 2000; Kumar *et al*., 2018). The newly obtained SSU rDNA sequences were then subjected to BLASTn search (NCBI) to identify similar sequences deposited in the GenBank database. For phylogenetic analyses, 67 SSU rDNA sequences from myxobolids were retrieved, encompassing all sequences with higher than 85% similarity to the isolates under study. This included all other heart infecting *Henneguya* spp. that have been sequenced to date, namely *Henneguya aegea* Katharios, Varvarigos, Keklikoglou, Ruetten, Sojan, Akter, Cascarano, Tsertou and Kokkari, 2020, *Henneguya akule* Work, Takata, Whipps and Kent, 2008, *Henneguya cynoscioni* Dyková, Buron, Roumillat and Fiala, 2011, *Henneguya lateolabracis* Yokoyama, Kawakami, Yasuda and Tanaka, 2003, *Henneguya mauritaniensis* Khlifa, Miller, Adlard, Faye and Sasal, 2012 and *Henneguya pagri* Yokoyama, Itoh and Tanaka, 2005. The only exception was the sequence of *Henneguya zikaweiensis* Sikama, 1938 (KR020026), which infects the heart of the freshwater cyprinid *Carassius auratus* (Linnaeus, 1758) but shares only 75.1% similarity with the isolates under study. The SSU rDNA sequence with GenBank accession no. OR699454 was selected as representative of the several sequences (OR699454, OR699456, OR699457, OR699458, OR699460 and OR699463) deposited in this database, belonging to an unnamed *Henneguya* sp. from *Chirodactylus variegatus* (Valenciennes, 1833) in Peru, sharing 91.4–91.6% similarity with the isolates under study. *Myxidium lieberkuehni* Bütschli, 1882 (X76638) and *Zschokkella auratis* Rocha, Casal, Rangel, Severino, Castro, Azevedo and Santos, 2013 (KC849425) were used as outgroup. Alignments were performed using MAFFT version 7, available online. Phylogenetic trees were obtained using both maximum-likelihood (ML) and Bayesian inference (BI) analyses. ML analysis was performed using the PhyML 3.0 program (Dereeper *et al*., 2008; Guindon *et al*., 2010) available at https://www.phylogeny.fr/. The general time reversible (GTR) model was selected based on the lowest score of the Bayesian information criterion and corrected Akaike information criterion, and bootstrap confidence values were calculated from 1000 replicates. BI analysis was performed in MrBayes v.3.2.6 (Ronquist and Huelsenbeck, 2003), with the GTR model with gamma-shaped rate variations across sites (Invgamma) (GTR + I + G) model evolution. Posterior probabilities were calculated using the Markov chain Monte Carlo method, with 4 chains running simultaneously for 1 million generations, burn-in set at 25% and trees sampled every 500 generations.

## Results

**Figure 1.**
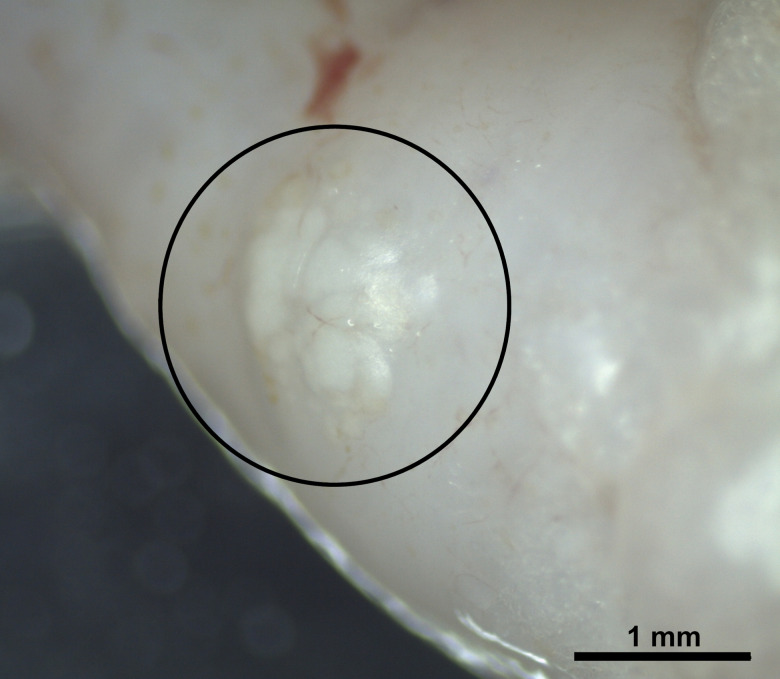
Irregular white plasmodia of *Henneguya cardii* n. sp. in the bulbus arteriosus (circle) of *Dicentrarchus labrax*.

Ninety-six specimens of *D. labrax* were examined. The sample consisted of 35 females, 29 males and 32 fish of undetermined sex, weighing 145.0 ± 72.5 (38.0–340.2) g, and measuring 22.5 ± 3.8 (14.7–30.6) cm in length. Thirteen specimens (13.5%), 4 females, 3 males and 6 of undetermined sex, presented myxosporean infection in both the heart (bulbus arteriosus) and spleen. Infected fish weighed 149.2 ± 49.6 (74.0–218.4) g and measured 23.2 ± 2.6 (19.0–26.7) cm in length. Numerous immature and mature myxospores could be observed within whitish irregularly shaped plasmodia, around 1 mm in size, located in the bulbus arteriosus (Fig. 1), while only loose mature myxospores were detected in fresh smears of the spleen. Morphological examination of myxospores and molecular analyses of the SSU rDNA gene identified the parasite as a new member of the genus *Henneguya*, herein described as *Henneguya cardii* n. sp.

### Taxonomic summary

#### Myxobolidae Thélohan, 1892 *Henneguya* Thélohan, 1892 *Henneguya cardii* n. sp.

Type-host: European seabass *Dicentrarchus labrax* (Linnaeus, 1758) (Moronidae).

Type-locality: Alvor estuary, Atlantic coast (37° 08′ N, 8° 37′ W), Portimão, Algarve, Portugal.

Site of infection: Bulbus arteriosus.

Prevalence: 13.5% (13 out of 96).

Type-material: Series of phototypes of the hapantotype, deposited together with a representative DNA sample in the Type Material Collection of the Laboratory of Animal Pathology, Interdisciplinary Centre of Marine and Environmental Research, Porto, Portugal, reference CIIMAR.2024.90.

Representative DNA sequences: Two SSU rDNA sequences (GenBank accession nos. ON119140 and ON119141).

Zoobank registration: LSID urn:lsid:zoobank.org:act:3E4A797A-D8D1-4119-85F9-E0C2B0ACE859.

Etymology: The specific name ‘*cardii*’ refers to the heart as the site of infection.

### Myxospore description

**Figure 2.**
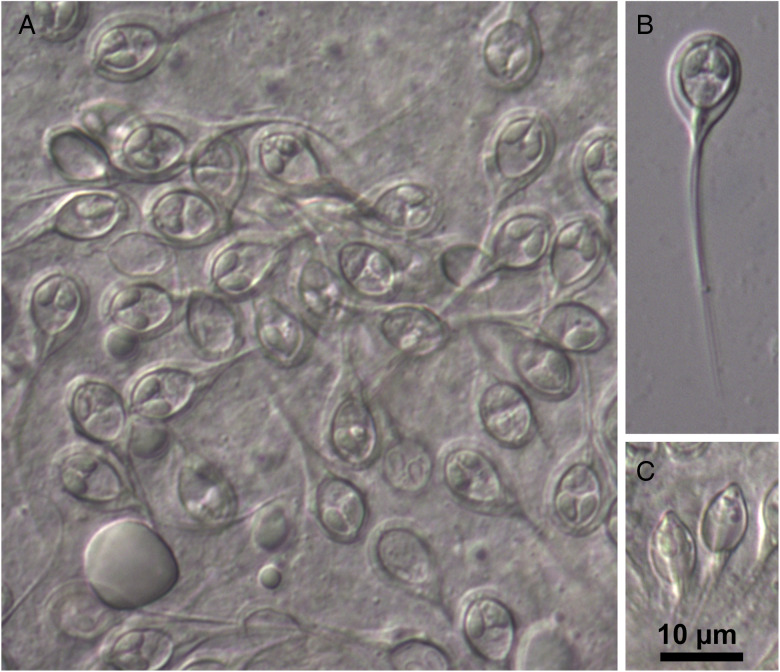
Mature myxospores of *H. cardii* n. sp. from the bulbus arteriosus of *D. labrax*: (A) myxospores from a ruptured plasmodium, (B) myxospore in valvular view and (C) myxospores in sutural view.

**Figure 3.**
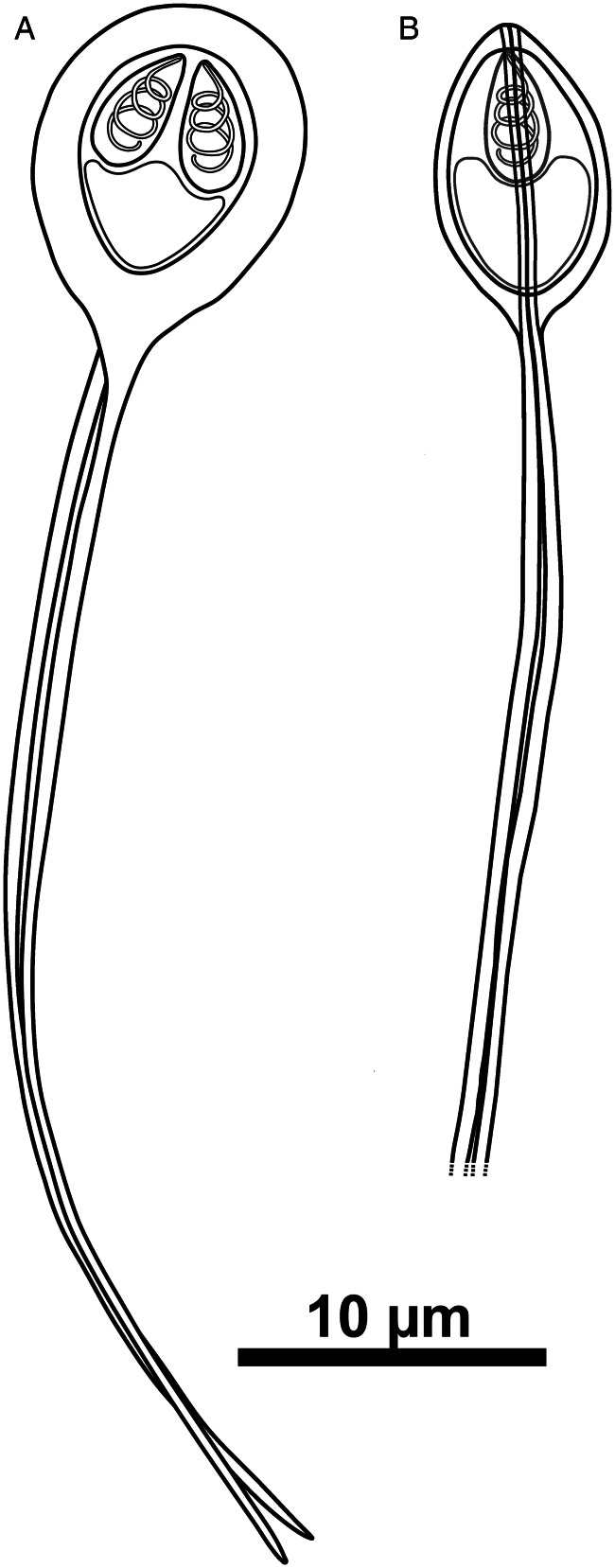
Line drawings of *H. cardii* n. sp. myxospores: (A) valvular view and (B) sutural view.

Myxospores broadly obovoid in frontal view and ellipsoidal in lateral view, with 2 equal caudal appendages forking slightly dislocated from the base. Suture line prominent. Two polar capsules, ovoid and symmetric, each comprising a polar tubule with 3–4 coils (Figs 2 and 3). Mature myxospores measuring 10.2 ± 0.6 (8.8–11.6) (*n* = 100) μm in length, 8.0 ± 0.7 (5.3–8.8) (*n* = 50) μm in width and 5.6 ± 0.2 (5.1–6.4) (*n* = 50) μm in thickness. Caudal appendages 36.6 ± 3.2 (27.4–42.9) (*n* = 28) μm long. Total spore length 47.6 ± 3.2 (41.2–53.2) (*n* = 20) μm. Polar capsules measuring 4.0 ± 0.2 (3.4–4.6) (*n* = 66) μm in length and 2.2 ± 0.1 (1.9–2.6) (*n* = 66) μm in width.

### Remarks

Myxospore morphometric variation was not detected between different isolates. Morphological comparison with other heart infecting *Henneguya* species revealed similarities to *Henneguya otolithi* Ganapati, 1941 and *H. lateolabracis* (see Table 2). However, *H. cardii* n. sp. differs from *H. otolithi* by being slightly shorter (8.8–11.6 vs 10.0–12.0 μm), thicker (5.1–6.4 vs 4.0–5.0 μm) and lacking a thickening running the myxospore body transversally (Ganapati, 1941). Despite some measures of *H. cardii* n. sp. overlapping with those of *H. lateolabracis* (Yokoyama *et al*., 2003), the SSU rDNA sequence of these species share only 87.4% similarity. Although *H. cardii* n. sp. shares similar myxospore shape with the unnamed *Henneguya* sp. previously reported from the heart of *D. labrax* in Sardinia (Culurgioni *et al*., 2010), morphometric comparison to this species is impossible due to the lack of measurements in the species report.

**Table 2.** Myxospore morphometry of *Henneguya* species infecting the heart, including the newly described *Henneguya cardii* n. sp.

Species	Type host	Type locality	SBL	SBW	SBT	PCS	PCL	PCW	PTC	CAL	TSL	Source
*H. cardii* n. sp.	*Dicentrarchus labrax*	Portugal	10.2 ± 0.6 (8.8–11.6)	8.0 ± 0.7 (5.3–8.8)	5.6 ± 0.2 (5.1–6.4)	=	4.0 ± 0.2 (3.4–4.6)	2.2 ± 0.1 (1.9–2.6)	3–4	36.6 ± 3.2 (27.4–42.9)	47.6 ± 3.2 (41.2–53.2)	Present study
*Henneguya aegea*	*Pagrus major*	Greece	12.8 ± 1.0 (10.0–14.9)	8.2 ± 1.1 (5.7–10.9)	6.2 ± 0.6 (5.2–7.0)	–	3.4 ± 0.6 (1.4–6.8)	1.7 ± 0.3 (0.8–2.5)	–	42.6 ± 6.5 (34.1–60.6)	64.9 ± 8.7 (53.6–82.8)	Katharios *et al*. (2020)
*Henneguya akule*	*Selar crumenophthalmus*	Hawaii	12.1 ± 0.8 (10.0–14.0)	7.4 ± 0.7 (5.0–9.0)	5.3 ± 0.6 (3.0–7.0)	–	3.4 ± 1.0 (2.0–6.0)	1.4 ± 0.5 (1.0–2.0)	3–4	–	40.8 ± 5.2 (29.0–52.0)	Work *et al*. (2008)
*Henneguya brachideuteri*	*Brachydeuterus auritus*	Senegal	11.5 (10.0–12.0)	8.3 (7.0–9.0)	–	=	4.3 (4.0–5.0)	2.6 (2.0–3.0)	–	26.9 (26.0–29.0)	37.1 (36.0–41.0)	Kpatcha *et al*. (1997)
*Henneguya cynoscioni*	*Cynoscion nebulosus*	USA	10.4 (9.8–11.7)	8.8	5.9	=	3.3	2.0	3–4	28.0 (23.5–33.3)	38.6 (34.3–44.1)	Dyková *et al*. (2011)
*Henneguya lateolabracis*	*Lateolabrax* sp.	Japan	10.7 (9.9–11.9)	7.5 (6.4–7.8)	6.2 (5.9–6.4)	=	3.4 (3.0–4.0)	1.7 (1.5–2.0)	3	37.7 (30.7–49.5)	–	Yokoyama *et al.* (2003)
*Henneguya mauritaniensis*	*Pagrus caeruleostictus*	Mauritania	12.3 ± 0.6	8.0 ± 0.4	–	=	4.1 ± 0.2	3.0	–	25.3 ± 3.2	17.9 (15.0–20.5)	Khlifa *et al.* (2012)
*Henneguya otolithi*	*Otolithes ruber*; *Pterotolithus maculatus*	India	10.0–12.0	6.0–8.5	4.0–5.0	–	3.0–4.0	2.0–2.5	5–6	35.0–40.0	–	Ganapati (1941)
*Henneguya ouakamensis*	*Mugil cephalus*	Senegal	10.9 ± 0.6 (9.0–13.0)	6.9 ± 0.4 (5.0–9.0)	–	=	3.8 ± 0.5 (3.0–4.0)	2.4 ± 0.4 (2.0–3.0)	–	9.9 ± 1.4 (6.0–14.0)	20.9 ± 1.5 (16.0–24.0)	Kpatcha *et al*. (1997)
*Henneguya pagri*	*P. major*	Japan	10.5 (9.9–11.9)	7.5 (6.4–8.4)	5.9 (5.4–6.4)	=	3.1 (2.5–4.0)	1.6 (1.5–2.0)	3	29.6 (24.8–34.7)	–	Yokoyama *et al*. (2005)
*Henneguya sebasta*	*Sebastes paucispinis*	USA	15.1 ± 1.3 (13.0–17.5)	9.2 ± 1.0 (5.6–11.0)	7.1 ± 1.0 (5.0–8.7)	–	4.5 ± 0.7 (3.7–5.6)	2.4 ± 0.4 (1.8–3.1)	4–6	62.0 ± 13.2 (32.5–87.5)	77.1 ± 12.8 (48.1–82.8)	Moser and Love (1975)
*Henneguya shackletoni*	*Eleginops maclovinus*	Falkland Islands	11.5 ± 1.5 (9.5–14.5)	8.5 ± 1.0 (7.0–11.0)	7.0 ± 1.0 (5.4–8.6)	=	3.5 ± 0.5 (3.0–5.0)	3.1 ± 0.5 (2.5–3.5)	6–7	37.0 ± 8.0 (25.0–51.0)	49.0 ± 9.0 (34.5–65.5)	Brickle *et al.* (2006)
*Henneguya visceralis*	*Electrophorus electricus*	South America	11.0–12.0	5.0–6.5	4.5	=	6.5–8.0	2.0	–	11.0–12.0	22.0–24.0	Jakowska and Nigrelli (1953)
*Henneguya vitiensis*	*Aurigequula fasciata*	Fiji Islands	13.7 (12.1–15.8)	7.8 (6.7–8.8)	–	–	3.2 (2.7–3.7)	1.7 (1.5–2.0)	3–4	29.1 (22.7–35.5)	–	Laird (1950)
*Henneguya yoffensis*	*P. caeruleostictus*	Senegal	13.3 (12.0–15.0)	9.1 (8.0–11.0)	–	=	3.4 (3.0–4.0)	2.3 (2.0–3.0)	–	32.1 (24.0–36.0)	46.1 (37.0–50.0)	Kpatcha *et al*. (1997)
*Henneguya zikawiensis*	*Carassius auratus*	Japan	9.5 (8.4–10.8)	8.0 (7.2–9.6)	6.0–6.6	–	4.5 (4.2–4.8)	3.0 (2.4–3.6)	4–5	15.0–38.4	23.4–49.2	Sikama (1938)

Measurements are given in micrometres. Means are provided with the standard deviation and the range in parentheses, when available.

CAL, caudal appendages length; PCL, polar capsule length; PCS, polar capsule size (=, polar capsules of equal size); PCW, polar capsule width; PTC, polar tubule coils; SBL, myxospore body length; SBT, myxospore body thickness; SBW, myxospore body width; TSL, total myxospore length; –, absence of data.

Overall, the species morphometrically more similar to *H. cardii* n. sp. are *Henneguya clini* Reed, Basson, Van As and Dyková, 2007, *Henneguya joalensis* Kpatcha, Faye, Diebakate, Fall and Toguebaye, 1997, *Henneguya lutjani* Kpatcha, Faye, Diebakate, Fall and Toguebaye, 1997 and *Henneguya tegidiensis* Nicholas and Jones, 1959. *Henneguya cardii* n. sp. and *H. clini* share nearly identical myxospore body length (10.2 vs 10.0 μm), body width (8.0 vs 7.9 μm), caudal appendage length (36.6 vs 34.0 μm), polar capsules size (4.0 × 2.2 vs 4.0 × 2.2 μm) and total myxospore length (47.6 vs 44.6 μm). However, these species differ in site of infection (heart vs gills), number of polar tubule coils (3–4 vs 4–5), host taxa (Moronidae vs Clinidae) and geographic distribution (limited to the North Atlantic vs limited to South Africa) (Reed *et al*., 2007). *Henneguya cardii* n. sp. differs from *H. joalensis* by having a slightly larger myxospore body (10.2 × 8.0 vs 8.9 × 6.3 μm) and smaller polar capsules (4.0 × 2.2 vs 4.7 × 2.5 μm) (see Kpatcha *et al*., 1997); from *H. lutjani* by having smaller myxospore body length (10.2 vs 11.6 μm), larger myxospore body width (8.0 vs 7.2 μm) and divergent polar capsules (4.0 × 2.2 vs 3.8 × 2.9 μm) (see Kpatcha *et al*., 1997) and from *H. tegidiensis* by being less thick (5.6 vs 6.7 μm) and having a distinct host environment (marine vs fresh water) (see Nicholas and Jones, 1959).

### Molecular analysis

Isolates R1H (ON119140) and R2H (ON119141) generated SSU rDNA sequences with 2016 and 2025 bp, respectively. These were 100% identical between each other, and BLASTn search did not retrieve highly similar SSU rDNA sequences from the GenBank database. The most similar sequences belonged to an unnamed *Henneguya* sp. (OR699454, OR699456, OR699457, OR699458, OR699460 and OR699463) from the Peruvian morwong *C. variegatus* (Valenciennes, 1833) (91.4–91.6%), *Henneguya lagunensis* de Azevedo, Negrelli, de Oliveira, Abdallah, Camara, Matos and Vieira, 2021 (MT676010) from Brazilian mojarra *Eugerres brasilianus* (Cuvier, 1830) (90.9%), *H. pagri* (AB183748) from red seabream *Pagrus major* (Temminck and Schlegel, 1843) (90.8%), *H. mauritaniensis* (JQ687060) from bluespotted seabream *Pagrus caeruleostictus* (Valenciennes, 1830) (90.7%) and *H. cynoscioni* (JN017203) from spotted weakfish *Cynoscion nebulosus* (Cuvier, 1830) (90.4%).

**Figure 4.**
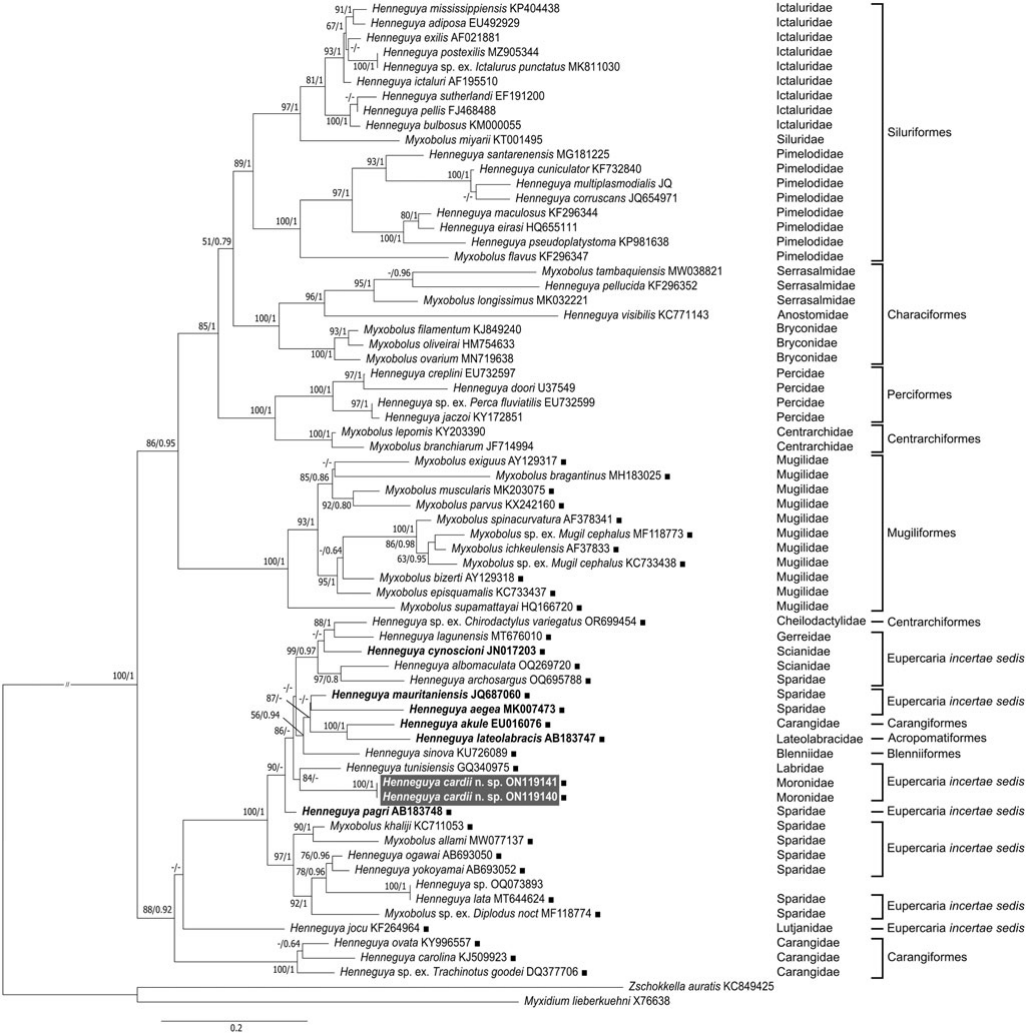
Tree topology resulting from the ML analysis of 65 SSU rDNA sequences from myxobolids, including *H. cardii* n. sp. (sequences within grey box), and all other marine heart-infecting *Henneguya* spp. (sequences in bold). Numbers at the nodes are ML bootstrap values/BI posterior probabilities; dashes represent a different branching of the BI tree or a bootstrap support value under 50. Species reported from brackish/marine environments are indicated by black squares (■). The remaining species were reported from freshwater environments. Information on host taxonomy is given on the right.

ML and BI analyses yielded similar topologies (Fig. 4). The isolates of *H. cardii* n. sp. clustered within a well-supported clade comprising other *Myxobolus* and *Henneguya* spp. that have also been reported from marine environments, and that mostly infect fish belonging to Eupercaria *incertae sedis*, but also Carangiformes, Blenniiformes, Acropomatiformes and Centrarchiformes. The sequences of other *Henneguya* spp. that have been reported from the heart were also grouped within this clade, namely *H. aegea*, *H. akule*, *H. cynoscioni*, *H. lateolabracis*, *H. mauritaniensis* and *H. pagri*. Another marine clade of myxobolids was retrieved and included solely the sequences of *Myxobolus* spp. reported from Mugiliformes. Overall, a host-associated phylogenetic pattern was retrieved in our analyses, with species that infect Siluriformes, Characiformes, Perciformes, Centrarchiformes, Mugiliformes, Carangiformes and Eupercaria *incertae sedis* mostly forming well-supported clades. Some entropy was retrieved within the clade comprising *H. cardii* n. sp. and other marine *Myxobolus*/*Henneguya*, but this is most likely the result of low inner nodal supports.

## Discussion

Currently, the description and taxonomic classification of myxosporeans rely on the combined analysis of several criteria, which include myxospore morphology, host and tissue specificity and molecular data (Atkinson *et al*., 2015). This holistic approach overcomes the artificiality of traditional morphology-based taxonomy, allowing reliable differentiation among congeners that share high morphological similarity. Nonetheless, the great majority of *Henneguya* species lack DNA data, making it inevitable to resort to myxospore morphology when identifying species that belong to this genus. The description of *H. cardii* n. sp. in this study was based on a comprehensive analysis of morphological, biological and molecular data. The species molecularly most similar to *H. cardii* n. sp. were an unnamed *Henneguya* sp. from the Peruvian morwong *C. variegatus*, *H. lagunensis*, *H. pagri*, *H. mauritaniensi*s and *H. cynoscioni*, ranging between 90.4 and 91.5% similarity. Thus, the smallest divergence to *H. cardii* n. sp. was 8.5%, meaning that *H. cardii* n. sp. is clearly distinct from GenBank records. All congeners without molecular data could be differentiated from *H. cardii* n. sp. based on morphological aspects and host species. An unnamed *Henneguya* sp. previously reported from the heart of *D. labrax* in Sardinia, Mediterranean Sea (Culurgioni *et al*., 2010), was observed to share some morphological similarity to *H. cardii* n. sp. Nonetheless, a formal comparison could not be performed due to the lack of myxospore measurements or molecular information from the Italian species report.

*Henneguya* spp. are typically parasites of fish gills but have also been described from several other organs, including the heart. The concomitant occurrence of *Henneguya* in the heart and other organs has been suggested to be the result of myxospore spreading through the bloodstream, as with *Henneguya visceralis*, which develops in the kidneys, disseminating *via* blood to the heart, liver, spleen, gallbladder, and other organs and tissues (Jakowska and Nigrelli, 1953). On the contrary, myxospores of *H. aegea* develop in the heart prior to being disseminated to other organs through the bloodstream (Katharios *et al*., 2020). The same was observed in *H. cardii* n. sp., which sporogonic development occurs in the heart, with mature myxospores being seemingly transported to the spleen, where they could be observed isolated.

The phylogenetic analyses performed in this study revealed *H. cardii* n. sp. positioned within a well-supported lineage of myxobolids that infect marine hosts, and that is predominantly comprised by *Henneguya* spp., including few *Myxobolus* representatives. The caudal appendages of *Henneguya* have been suggested to optimize the dispersion capacity of myxospores in freshwater habitats or over longer distances (Liu *et al*., 2019). Thus, the predominance of the *Henneguya* morphotype in the marine lineage of myxobolids strengthens the contention that character evolution is a complex and dynamic process that cannot be fully comprehended based on a single criterion, given that it most likely involves several host- and environment-related factors. For instance, mugiliform-infecting myxobolids mostly belong to the genus *Myxobolus*, whose morphotype has been hypothesized to optimize transmission to the deposit-feeding oligochaetes that host their actinospore development in marine habitats (Rocha *et al*., 2020). Discover of additional Myxobolidae life cycles would allow a better understanding of myxospore morphological character evolution.

In agreement with previously published cladograms of myxobolids (Carriero *et al*., 2013; Liu *et al*., 2019), our phylogenetic analyses retrieved host-associated clustering. A tendency of gill and muscle-infecting myxobolids to cluster according to the site of infection has also been shown in the literature (Eszterbauer, 2004; Molnár and Eszterbauer, 2015) and suggests tissue tropism as a potential minor evolutionary driver for this myxosporean group. Accordingly, the tree topology obtained in this study supports a close relationship between marine heart-infecting *Henneguya* species. The overall low support of the marine lineage inner nodes suggests that a hidden diversity of marine myxobolids is yet to be explored that could ultimately provide better resolution of large- and fine-scale factors influencing evolutionary patterns.

Although clinical signs of infection by *H. cardii* n. sp. were not observed in this study, a histopathological assessment is required to evaluate the potential pathological impact that this myxosporean might have in the production of European seabass.

## Data Availability

All data supporting the findings of this study are available within the paper. Sequence data are available on the NCBI GenBank database.
